# Increased substrate stiffness disrupts nuclear-cytoskeletal mechanical coupling in senescent cells

**DOI:** 10.1016/j.mtbio.2025.102472

**Published:** 2025-10-27

**Authors:** Mina Sohrabi Molina, Erik Brauer, Rebecca Günther, Stephanie Diederich, Ansgar Petersen

**Affiliations:** a Berlin Institute of Health at Charité – Universitätsmedizin Berlin, BIH Center for Regenerative Therapies, Germany; b Berlin School for Regenerative Therapies, Charité – Universitätsmedizin Berlin, Germany; c Berlin Institute of Health at Charité – Universitätsmedizin Berlin, Julius Wolff Institute – Center for Musculoskeletal Biomechanics and Regeneration, Germany

**Keywords:** Senescence, Mechano-adaptation, Substrate stiffness, Nuclear mechano-adaptation, Nuclear-cytoskeletal coupling

## Abstract

The physical coupling between the nucleus and the cytoskeleton is essential for the mechanobiological adaptation of cells to mechanical cues presented by the surrounding extracellular matrix (ECM). Although aging is known to influence both cellular and ECM mechanical properties, it remains poorly understood how cellular senescence, a hallmark of aging, affects cellular mechano-adaptation. Here, we use substrate stiffness as a mechano-modulatory cue across three distinct models of senescence and demonstrate that senescent fibroblasts are limited in their capacity to integrate mechanical signals of increasing stiffness. The senescent nucleus undergoes progressive actin-mediated deformation and flattening as substrate stiffness increases, until a mechanical threshold is reached that provokes a decoupling of the nucleus from the cytoskeleton. This mechanical disengagement of the nucleus on stiff substrates is accompanied by a loss of cytoskeletal organization, abnormal focal adhesion (FA) maturation, and nuclear softening. We further suggest that the loss of nuclear compression is linked to changes in the nuclear localization of the key mechanosensitive transcriptional regulator Yes-associated protein (YAP). Our findings reveal a fundamental biophysical limitation in the mechano-adaptive response of senescent cells to high-stiffness environments, conditions typically associated with advanced tissue maturation and pathological scarring, which may underlie altered nuclear mechanotransduction and contribute to their specific role in both physiological and pathological contexts.

## Graphical abstract

**Figure:**
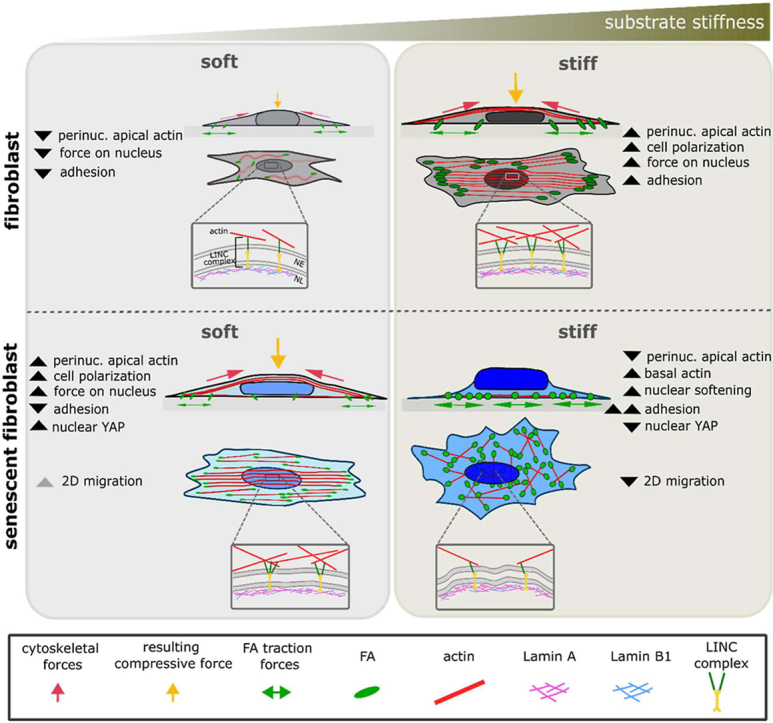

## 1 Introduction

The ability of tissue-resident cells to sense and adapt to mechanical cues from their microenvironment is essential for physiological cell function. A key player in this adaptation is the physical coupling between the cytoskeleton and the nucleus, which enables cells to respond to external forces and maintain mechanical homeostasis [[1], [2], [3], [4]]. Whether responding to externally applied mechanical stresses, tight pores within the extracellular matrix (ECM), or changes in ECM stiffness, cells must adjust their cytoskeletal and nuclear structures to maintain their intrinsic tensional homeostasis [[5], [6], [7], [8]]. While it is well known that ECM composition, structure, and mechanical properties change with age, leading to large-scale adaptations at the tissue level [9,10], the response of aging cells to mechanical alterations of the environment remains poorly understood.

A key determinant of cellular mechano-adaptation is the stiffness of the ECM, which is sensed by cells through the application of tensional forces [6,11]. Adjusting cellular and nuclear components to such mechanical inputs regulates numerous cell functions, such as adhesion, migration, and ultimately, cell fate [[12], [13], [14], [15]]. Here, the dynamic re-arrangement of actin filaments (F-actin) induces changes in cellular shape and generates mechanical stresses to both the ECM and the cell's nucleus [6,14,16]. Through its connection to the nuclear envelope (NE) via the linker of nucleoskeleton and cytoskeleton (LINC) complex, the F-actin cytoskeleton not only transmits mechanical cues that regulate gene expression [17], but also dampens forces to protect the NE and the underlining chromatin [18,19]. At the same time, cytoskeletal tension influences nuclear pore dynamics and the translocation of mechanosensitive regulators such as Yes-associated protein (YAP), linking nuclear compression to nuclear mechanotransduction [20]. Further, nuclear-cytoskeletal coupling facilitates nuclear movement and positioning during migration, particularly through confined 3D microenvironments [21]. Therefore, beyond force transmission, nuclear-cytoskeletal coupling serves multiple roles in maintaining nuclear integrity, enabling nuclear repositioning, and regulating mechanosensitive signaling.

Central to the process of aging is the accumulation of senescent cells [22] that arise through proliferation-induced telomere attrition or direct DNA damage [[23], [24], [25]]. Senescent cells are characterized by an irreversible cell cycle arrest and secretory phenotype which alters the ECM composition and the stiffness of the microenvironment [[26], [27], [28]]. Beyond their secretory profile, senescent cells also undergo a myriad of biophysical changes, including strong morphological and cytoskeletal alterations, influencing cell mechanics and migration [[29], [30], [31]]. Indeed, we recently demonstrated across multiple models of senescence that senescent fibroblasts consistently exhibit stronger single cell forces and adhesion, with functional differences in 3D contraction within an *in vitro* tissue model [31]. At the nuclear level, structural changes are caused by alterations in nuclear lamin proteins of the NE [32,33]. The loss of Lamin B1 or mutations in Lamin A/C are hallmarks of senescent cells and premature aging phenotypes that compromise NE stability [[34], [35], [36]]. Alterations of other NE components, such as the LINC complex, have also been associated with senescence [[37], [38], [39]]. While the biophysical changes associated with senescent cells are evident, the consequences of such changes on the mechano-adaptation of senescent cells is less understood. Emerging evidence suggests that senescent cells may perceive exogenous mechanical forces differently, with consequences for both their phenotype and nuclear mechanics [[39], [40], [41]]. However, the extent to which senescent cells coordinate cytoskeletal and nuclear adaptations in response to mechanical changes remains unknown.

In this study, we investigated how senescence alters the cellular mechano-adaptation to changes in substrate stiffness by culturing cells on soft (1.7 kPa), intermediate-stiff (48.3 kPa) hydrogels and rigid tissue culture plastic (E ∼ 1 GPa) substrates. Three distinct methods of inducing senescence were applied to human dermal fibroblasts (hdFs) creating a comprehensive model system of cellular senescence in tissue-forming cells. Our findings reveal that cellular senescence leads to a strong shift in cellular mechano-adaptation, such that high-stiffness environments provoke abnormal cell morphology, actin cytoskeletal organization, FA maturation, and changes in nuclear mechanics. Notably, we show that senescent cells cope with increasing stiffness through nuclear softening and disruption of the mechanical link between the actin cytoskeleton and the nucleus, accompanied by alterations in the nuclear localization of the mechanosensitive transcriptional co-activator YAP. Our work highlights the inability of senescent cells to properly integrate mechanical cues associated with advanced tissue healing and maturation, providing novel insights into the biophysical challenges that may shape their behavior in aging and disease, while also pointing to a broader impairment of nuclear mechanotransduction.

## 2 Results

### 2.1 Validating different forms of cellular senescence on human dermal fibroblasts *in vitro*

**Fig. 1:**
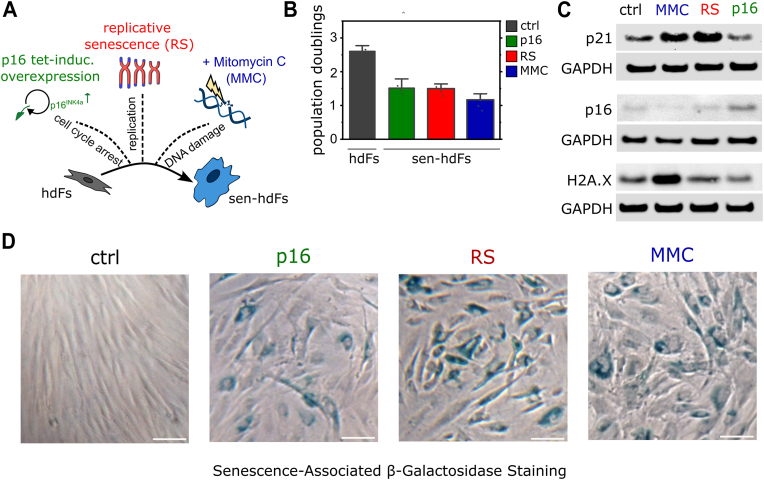
***In vitro* validation of cellular senescence in human dermal fibroblasts. (A)** Schematic showing senescence induction mechanism in hdFs via three independent stimuli: (1) over-expression of p16 through a tetracycline-inducible construct enforcing a cell cycle arrest, (2) long-term cell culture leading to telomere shorting resulting in replicative senescence (RS), and (3) treatment with DNA crosslinking agent Mitomycin C (MMC) causing DNA damage. **(B)** Reduced proliferation of senescent hdFs (p16, RS, and MMC) expressed as population doublings. **(C)** Immunoblotting of cell cycle inhibitors p21Cip and p16Ink4a, and DNA damage marker phospho-H2A.X in hdFs and senescent hdFs (p16, RS, and MMC). **(D)** Representative phase-contrast images of hdFs (ctrl) and senescent hdFs (p16, RS and MMC) cultured for 14 days showing increased cellular morphology and increased senescence-associated-β-galactosidase activity (blue staining) in senescent hdFs. Scale bar 100 μm. (For interpretation of the references to color in this figure legend, the reader is referred to the Web version of this article.)

Cellular senescence is a heterogeneous process, with its phenotype shaped by the specific stressor [42]. In our previous study [43], we comprehensively characterized senescence models using an extended panel of markers and showed that functional differences between the senescent models were most pronounced between DNA damage-dependent and -independent states, rather than between specific pathways. Building on this foundation, in the present study we assessed how substrate stiffness influences distinct forms of senescence by establishing three distinct models: (1) cell cycle arrest-mediated (DNA damage-independent) (2) telomere-dependent (proliferation exhaustion), and (3) DNA damage-mediated (genotoxic-stress) senescence. Primary hdFs were driven into senescence through (1) conditional over-expression of the cyclin-dependent kinase inhibitor CDKN2a/p16 via a tetracycline-inducible construct [43] (group p16), (2) long-term culture leading to proliferation exhaustion resulting in replicative senescence (group RS), or (3) treated with the DNA crosslinking agent Mitomycin C (group MMC) (Fig. 1a). Cell proliferation was reduced in all three treated groups when compared to control hdFs (Fig. 1b and Fig. S1a). Western blot analysis further demonstrated an increase in CDKN1a/p21 cyclin-dependent kinase inhibitor in telomere-dependent (RS) and DNA-damage-mediated (MMC) senescent groups; whereas cell cycle arrest-mediated senescent cells (p16) showed an increase in p16 protein expression (Fig. 1c and Fig. S1d and e). Protein analysis of H2A.X phosphorylation, a DNA damage marker, showed an increase in MMC-treated cells (Fig. 1c and Fig. S1c). Lastly, we observed an increase of senescence-associated-β-galactosidase activity, a commonly described marker for senescence, for all treated groups (Fig. 1d). These results confirm the induction of cellular senescence across all three approaches, thereby establishing three independent models of senescence, each representing a key feature of the program: (1) cell cycle arrest, (2) proliferative exhaustion, and (3) DNA damage.

### 2.2 Altered stiffness-mediated mechano-adaptation drives specific cellular morphologies in senescent cells

Cell spreading and the resulting cell shape are key indicators of cellular function and are strongly influenced by microenvironmental stiffness [44]. To explore morphological changes of senescent hdFs (sen-hdFs) in response to microenvironmental stiffness, we either used polyacrylamide (PA) hydrogels with variable stiffness or tissue culture plastic (TCP) as cell culture substrates. Both, PA hydrogels and TCP were coated with collagen to represent physiological and supra-physiological stiffness conditions, respectively. We used PA-hydrogels as a modulator of mechano-adaptation, due to their controlled mechanical properties, which efficiently modulate cell structure and mechanics [45]. Thus, hdFs and sen-hdFs were cultured for one day on soft (E = 1.7 kPa), intermediate-stiff (E = 48.3 kPa) PA hydrogels, or on rigid TCP plates (E ∼ 1 GPa). The soft and intermediate stiffness values of 1.7 kPa and 48.3 kPa were selected to fall within the range of tissue stiffnesses observed during tissue regeneration and wound healing, spanning from soft hematoma (∼2 kPa) [46] to more mature, stiffer granulation tissue (∼50 kPa) [47].

**Fig. 2:**
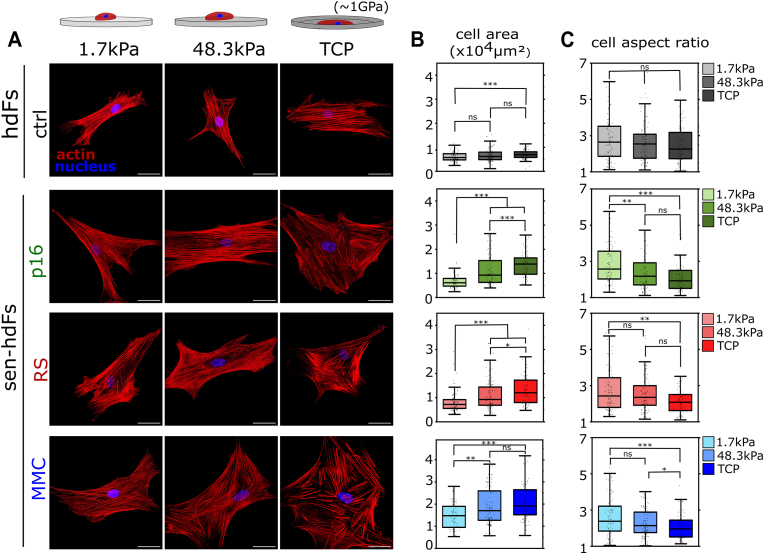
**Enhanced cell morphological alterations of senescent hdFs in response to stiffness-varying substrates. (A)** Representative immunostained images of hdFs (ctrl) and senescent hdFs (p16, RS and MMC) cultured for one day on collagen-coated stiffness-varying substrates: soft (E = 1.7 kPa), intermediate-stiff (E = 48.3 kPa), and rigid TCP (E ∼ 1 GPa). Cells were stained for actin cytoskeleton (red) and cell nuclei (blue). **(B)** Quantification of changes in cell area and **(C)** cell aspect ratio in response to stiffness-varying substrates. Each data point represents an individual cell (N ≥ 60 cells) from at least 3 independent experiment. Significance levels indicate: ∗p < 0.05, ∗∗p < 0.01, and ∗∗∗p < 0.001. Scale bar 50 μm. (For interpretation of the references to color in this figure legend, the reader is referred to the Web version of this article.)

F-actin staining revealed that the cell area increased with increasing substrate stiffness across all groups, while cell spreading was generally more pronounced in all three senescence models (Fig. 2a). Quantitative analysis revealed that, from soft (E = 1.7 kPa) to rigid TCP substrates, median cell area increased from 0.66 to 1.34 × 10^4^ μm^2^ (2.03-fold), 0.72 to 1.22 × 10^4^ μm^2^ (1.69-fold), and 1.47 to 2.05 × 10^4^ μm^2^ (1.39-fold) for senescent groups p16, RS, and MMC, respectively; compared to an increase from 0.57 to 0.69 × 10^4^ μm^2^ (1.21-fold) in control hdFs (Fig. 2b and Table S1). In agreement with our observation that cell morphology in sen-hdFs changes from a spindle shape on soft substrates to a pancake-like morphology on TCP, all three models of sen-hdFs exhibited a clear decrease in morphological aspect ratio in response to increasing substrate stiffness. Quantification showed that, from soft to TCP substrates, median aspect ratio decreased from 2.58 to 1.93 (1.34-fold), 2.63 to 2.08 (1.26-fold), and 2.58 to 1.96 (1.32-fold) for sen-hdFs groups p16, RS, and MMC groups, respectively (Fig. 2c and Table S2). In contrast, no statistically significant difference in the aspect ratio of control hdFs was detected in response to increasing stiffness. Together, these findings highlight how substrate stiffness strongly influences sen-hdFs morphology. In constrast to hdFs, sen-hdFs showed dramatic morphological changes with increasing substrate stiffness. Interestingly, the elongated shape found for sen-hdFs on soft substrates, was quite similar to the shape of hdFs and deviated clearly from the commonly reported pancake-like, senescent morphology. Together, these findings reveal a shift in the mechano-adaptation process, with sen-hdFs displaying hdF-like morphology on soft substrates and strong morphological changes on medium to high-stiffness substrates.

### 2.3 High-stiffness substrates disrupt coordinated actin fiber alignment in senescent cells

**Fig. 3:**
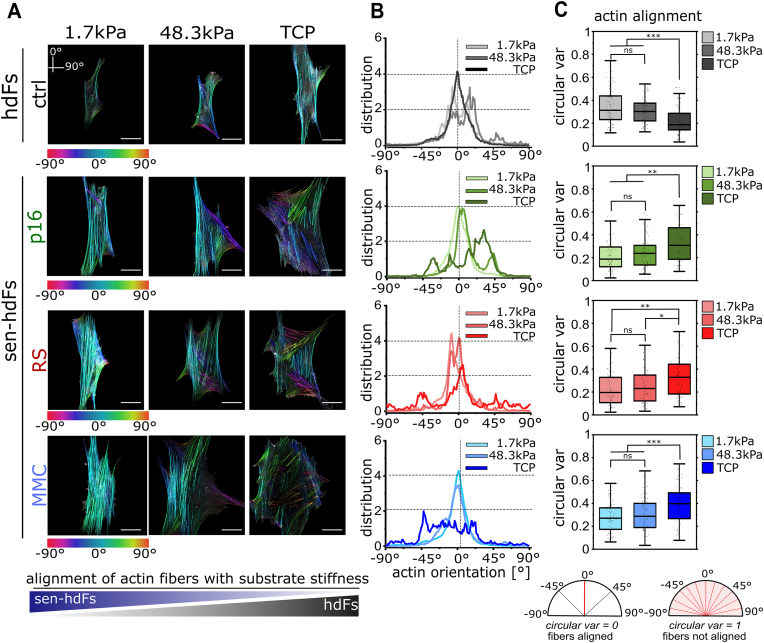
**Altered actin cytoskeletal re-arrangement of senescent hdFs in response to stiffness-varying substrates**. **(A)** Representative orientation-colored images showing local orientation of actin fibers of hdFs (ctrl) and senescent hdFs (p16, RS and MMC) cultured for one day on collagen-coated stiffness-varying substrates: soft (E = 1.7 kPa), intermediate-stiff (E = 48.3 kPa), and rigid TCP (E ∼ 1 GPa). The different colors indicate different orientations of actin fibers according to the color scale with respect to the cell axis (0°). **(B)** Corresponding distribution plots of the actin orientation-colored images for the different stiffness within each cell group. **(C)** Box plots showing the circular variance of actin fiber distribution in individual cells, with lower values indicating highly aligned fibers and higher values indicating increased fiber isotropy and disorganization. Each data point represents an individual cell (N ≥ 60 cells) from at least 3 independent experiments. Significance levels indicate: ∗p < 0.05, ∗∗p < 0.01, and ∗∗∗p < 0.001. Scale bar 50 μm. (For interpretation of the references to color in this figure legend, the reader is referred to the Web version of this article.)

The actin cytoskeleton is a mechano-adaptive component that plays a crucial role in determining cell shape and function [11]. To explore the cytoskeletal mechanisms behind the strong stiffness-mediated morphological changes in sen-hdFs, we examined how substrate stiffness modulates their actin cytoskeletal re-organization. Cytoskeleton filament descriptors, such as fiber orientation, provide a quantitative indication for cellular mechano-adaptation [48]. Therefore, we quantified the orientation of cytoskeletal actin fibers from immunostained images relative to the cell axis (Fig. 3a and b). We used circular variance (CVar) as a quantitative measure of actin fiber organization which was calculated based on clustering of fiber orientation angles within individual cells. CVar values range from 0 (highly aligned actin fibers) to 1 (isotropic actin fiber distribution) (Fig. 3c).

On soft (E = 1.7 kPa) and intermediate-stiff(E = 48.3 kPa) substrates, control hdFs exhibited a diffused actin pattern, resulting in a median CVar value of 0.31 (Q1: 0.23, Q3: 0.44) and 0.30 (Q1: 0.22, Q3: 0.38), respectively. In contrast, on high-stiffness TCP (E ∼ 1 GPa), hdFs formed well-developed, parallel actin stress fibers, leading to a lower CVar of 0.19 (Q1: 0.14, Q3: 0.29). Strikingly, all three senescent groups on soft and intermediate-stiff substrates adopted an F-actin organization that closely resembled the well-aligned stress fibers observed in control hdFs on stiff TCP. The well-defined, highly aligned actin stress fibers were associated with median CVar values ranging from 0.22 to 0.29 on soft substrates (Q1: 0.11–0.17, Q3: 0.30–0.36) and from 0.24 to 0.30 on intermediate-stiff substrates (Q1: 0.12–0.19, Q3: 0.31–0.40). In contrast, on rigid TCP, sen-hdFs exhibited disrupted F-actin alignment, characterized by decreased actin fiber ordering (CVar = 0.33–0.40; Q1: 0.18–0.27, Q3: 0.44–0.49) and the presence of short, thick stress fibers arranged in domains with differing orientations. These findings demonstrate that substrate stiffness significantly modulates the cytoskeletal organization of sen-hdFs, not only revealing an opposing pattern of F-actin remodeling compared to hdFs but also suggesting a shift in the stiffness-mediated actin re-arrangement, where sen-hdFs exhibit cytoskeletal features on soft substrates that are typically observed in hdFs at high-stiffness conditions.

### 2.4 Senescent cells exhibit an enhanced stiffness-mediated FA maturation and a distinct FA spatial distribution

**Fig. 4:**
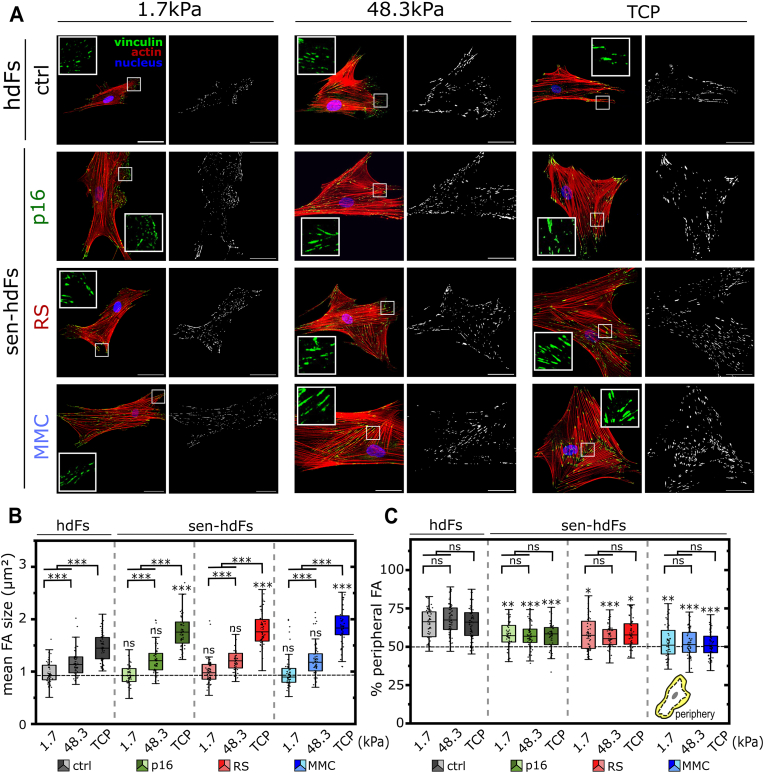
**Enhanced maturation and altered spatial distribution of focal adhesions of senescent hdFs in response to stiffness-varying substrates**. **(A)** Representative immunostained images (left) and corresponding inverted binary images (right) showing focal adhesions (FAs) of hdFs (ctrl) and senescent hdFs (p16, RS and MMC) cultured for one day on collagen-coated stiffness-varying substrates: soft (E = 1.7 kPa), intermediate-stiff (E = 48.3 kPa), and rigid TCP (E ∼ 1 GPa). Cells were stained for actin cytoskeleton (red), cell nuclei (blue) and vinculin (green). **(B)** Quantification of mean FA size per cell and **(C)** percentage of FAs located at the periphery of the cell. Each data point represents an individual cell (N ≥ 50 cells) from 3 independent experiments. Significance levels indicate: ∗p < 0.05, ∗∗p < 0.01, and ∗∗∗p < 0.001. For comparisons within same cell group, significance is indicated using asterisks accompanied by connecting lines. For comparisons between control cells (hdFs) and senescent hdFs under the same substrate condition, significance is indicated by asterisks placed directly above the box. Scale bar 50 μm. (For interpretation of the references to color in this figure legend, the reader is referred to the Web version of this article.)

The actin cytoskeleton is closely linked to integrin-based transmembrane complexes, known as FAs, which connect the cell to its ECM and serve as sites for force transmission and mechano-sensing [15]. Since mechanical signals are transduced through FAs to the cytoskeleton, we hypothesized that the pronounced changes in cell morphology and cytoskeletal re-organization of sen-hdFs are associated with an altered FA maturation. We thus analyzed FA maturation and FA shape (elongation) in response to stiffness through visualization of the FA marker protein vinculin. Consistent with previous findings [15,49], maturation of FAs, indicated by an increase in mean FA size, correlated with increased substrate stiffness in all groups (Fig. 4a and b). Notably, no significant difference in mean FA size was observed between control hdFs and sen-hdFs in neither soft (E = 1.7 kPa) or intermediate-stiff (E = 48.3 kPa) substrates. However, on rigid TCP substrates (E ∼ 1 Gpa), sen-hdFs exhibited a significantly greater increase in mean FA size compared to their non-senescent counterpart, consistent with our previous findings [43]. While FA size in hdFs reached a median of 1.4 μm^2^ (Q1: 1.2, Q3: 1.6) on TCP, FA size in sen-hdFs on TCP exceeded a median of 1.8 μm^2^ (Q1: 1.5–1.7, Q3: 1.9–2.1). Quantitative analysis revealed almost a 1.5-fold increase in the mean FA size of hdFs on soft compared to TCP substrates, whereas the mean FA size of sen-hdFs under the same conditions was increased by 1.9-, 2-, and 2.1-fold, for p16, RS, and MMC groups, respectively (Fig. 4a and b and Table S3). Moreover, FA elongation, quantified as the mean FA aspect ratio (AR) per cell, showed a lower fold change with increasing stiffness in all senescent groups compared to their non-senescent counterpart (Fig. S2b), as FAs of sen-hdFs were already more elongated on softer substrates. This effect was most clearly visible for RS and MMC groups where the mean FA AR on soft (E = 1.7 kPa) substrates was 2.1 (Q1: 2.1, Q3: 2.3) and 2.2 (Q1: 2.1, Q3: 2.4), respectively, whereas it was only 1.9 (Q1: 1.8, Q3: 1.9) for control cells.

The difference in cytoskeletal arrangement associated with senescence also motivated a closer look at the spatial distribution of FAs in senescent cells. To assess spatial FA distribution, we quantified the percentage of all FAs that were located at the cell's periphery. Independent of substrate stiffness, the vast majority of FAs ( ≥ 65 %) in control hdFs were located at the cell periphery (Fig. 4c). In contrast, FAs of sen-hdFs showed a homogenous spatial FA distribution with less peripheral FAs (51–58 %) and therefore more FAs across the total cell body. Particularly notable was the high abundance of very large FAs distributed throughout the cell body of senescent cells on TCP, as opposed to the peripheral localization of large FAs in hdFs on such plastic substrates. This feature was especially prominent in DNA-damage induced (MMC) senescent cells (Fig. S2c). Taken together, these findings not only demonstrate that the senescence program strongly modulates the spatial distribution of FA sites but also that rigid substrates (i.e. TCP) in particular provoke a strikingly strong maturation of FAs in senescence.

**Fig. 5:**
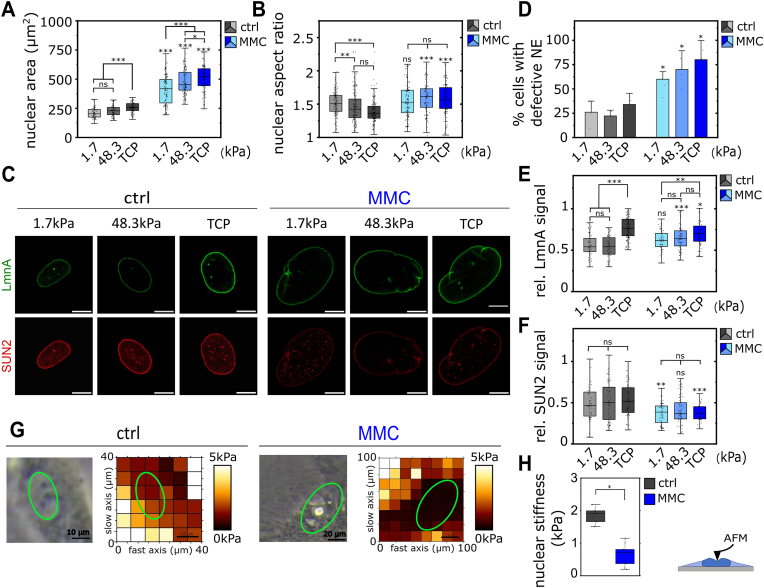
**Alterations in nuclear mechanics andnuclear-cytoskeletalcoupling in senescent hdFs. (A)** Quantification of changes in nuclear area and **(B)** nuclear aspect ratio of hdFs (ctrl) and senescent hdFs (MMC) cultured for one day on collagen-coated stiffness-varying substrates: soft (E = 1.7 kPa), intermediate-stiff (E = 48.3 kPa), and rigid TCP (E ∼ 1 GPa). **(C)** Representative immunofluorescence staining of LMNA (green) and SUN2 (red) in the nuclear envelope of hdFs (ctrl) and senescent hdFs (MMC). **(D)** Quantification of percentage of cells with abnormal nuclear envelope in hdFs and senescent hdFs cultured on stiffness-varying substrates. Each data point represents an individual experiment (N = 5–6) in which at least 30 nuclei where analyzed per condition. **(E)** Quantification of relative nuclear envelope LMNA signal and **(F)** SUN2 signal in response to stiffness-varying substrates. Each data point represents an individual cell (N ≥ 60) from at least three independent experiments. **(G)**. Representative phase-contrast AFM images (left) of probed hdF (ctrl) and senescent hdFs (MMC) cultured on collagen-coated TCP and corresponding Young's Modulus heatmap of the probed region (right). Green line indicates the cell's nucleus. **(H)** Quantification of Young's Modulus of the nucleus of hdFs (ctrl) and senescent hdFs (MMC) cultured for one day on collagen-coated TCP. Each data point represents an individual cell (N ≥ 7) cells from 2 independent AFM experiments. Significance levels indicate: ∗p < 0.05, ∗∗p < 0.01, and ∗∗∗p < 0.001. For comparisons within same cell group, significance is indicated using asterisks accompanied by connecting lines. For comparisons between control cells (hdFs) and senescent hdFs (MMC) under the same substrate condition, significance is indicated by asterisks placed directly above the box. Scale bar 10 μm unless otherwise indicated. (For interpretation of the references to color in this figure legend, the reader is referred to the Web version of this article.)

**Fig. 6:**
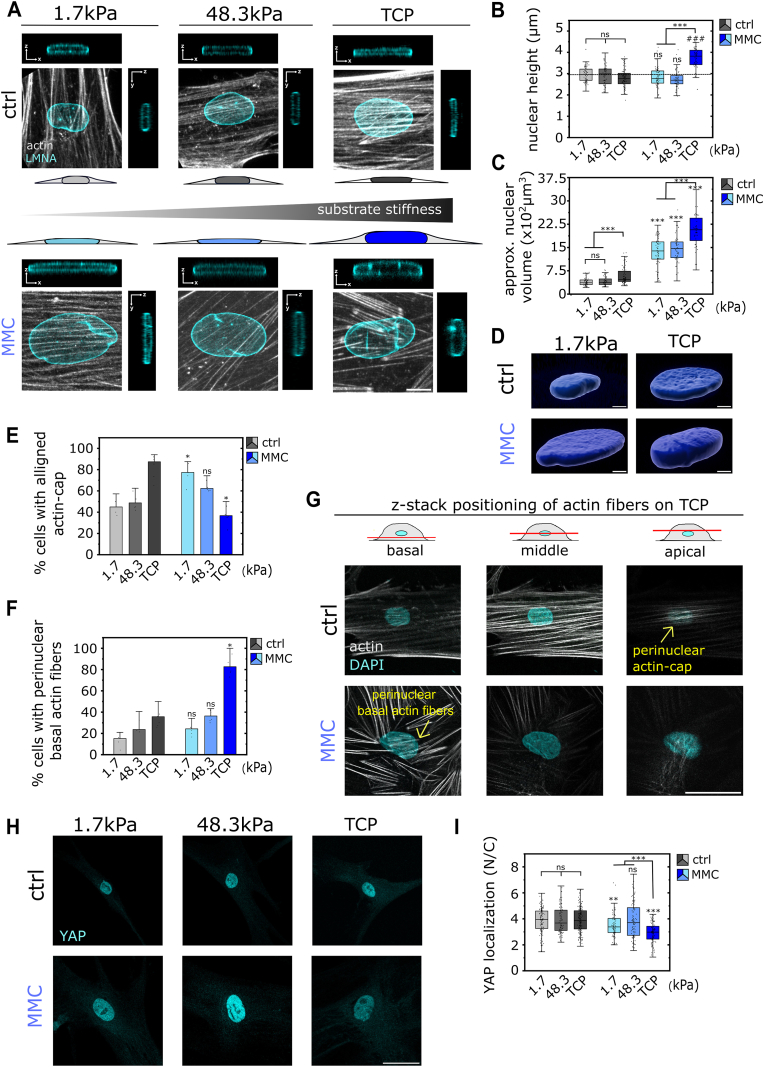
**Stiffness-mediated disruption of perinuclear actin-cap and loss of nuclear flattening in senescent cells, accompanied by altered nuclear YAP localization. (A)** Representative immunostained images of the nuclei of hdFs (ctrl) and senescent hdFs (MMC) cultured for one day on collagen-coated stiffness-varying substrates: soft (E = 1.7 kPa), intermediate-stiff (E = 48.3 kPa), and rigid TCP (E ∼ 1 GPa). Corresponding reconstructed x-z (top) and y-z (right) views from confocal z-stack images showing nuclear side views. Cells were stained for actin (grey) and LMNA (cyan). **(B)** Quantification of nuclear height and **(C)** approximate nuclear volume. **(D)** Representative 3D reconstruction of the nuclear volume of hdFs (top) and senescent hdFs (bottom) cultured on soft (left) and rigid TCP (right) substrates**. (E)** Quantification of percentage of cells with organized perinuclear actin fibers and **(F)** prominent basal perinuclear actin fibers in hdFs and senescent hdFs (MMC) cultured on stiffness-varying substrates. Each data point represents an individual experiment (N ≥ 6) in which at least 30 nuclei where analyzed per condition. **(G)** Representative immunostained images of hdFs (ctrl) and senescent hdFs (MMC) cultured on collagen-coated rigid TCP (E ∼ 1 GPa) showing confocal sections of the actin fiber network around the perinuclear area at the basal (left), mid-height (middle), and apical surface (right). Apical surface (right) displays prominent organization of actin fibers in hdFs and lack of actin cap formation in senescent hdFs (MMC). Basal surface (left) displays prominent disorganized perinuclear actin fibers in senescent hdFs (MMC). **(H)** Representative immunostained images of hdFs (ctrl) and senescent hdFs (MMC) cultured on the stiffness-varying substrates. Cells were stained for YAP (cyan). **(I)** Quantification of Nuclear to Cytoplasmic (N/C) YAP ratio. Each data point represents an individual cell (N ≥ 60 cells) from 3 independent experiments Significance levels indicate: ∗p < 0.05, ∗∗p < 0.01, and ∗∗∗p < 0.001. For comparisons within same cell group, significance is indicated using asterisks accompanied by connecting lines. For comparisons between control cells (hdFs) and senescent hdFs (MMC) under the same substrate condition, significance is indicated by asterisks placed directly above the box. Scale bars: 10 μm for **(A)** and 50 μm for **(G)** and **(H).** (For interpretation of the references to color in this figure legend, the reader is referred to the Web version of this article.)

As demonstrated above, RS and p16 senescent groups exhibit similar, although less pronounced, stiffness-mediated mechano-adaptation compared to the MMC group. Thus, for clarity, only the data of the MMC group, representing the late-stage (deep) senescence phenotype, is shown in the following main figures (Fig. 5, Fig. 6). Data for RS and p16 groups are included in the supplementary material. “Sen-hdFs” in the following sections refers to MMC-treated cells.

### 2.5 Are senescence-associated changes in cytoskeletal organization and FAs linked to alterations in nuclear mechanics?

Nuclear mechano-adaptation relies on a dynamic balance between cytoskeletal forces and nuclear mechanical properties [50]. Due to the exaggerated response of the actin cytoskeleton and FA complexes to substrate stiffness in sen-hdFs, we were interested in the related nuclear adaptations. In senescent cells, the nucleus undergoes strong morphological and structural changes [33]. Based on this understanding, we first analyzed nuclear morphology in response to stiffness (Fig. 5 and Fig. S3). We observed a strong increase in nuclear area in sen-hdFs compared to control hdFs on TCP (Fig. 5a and Fig. S3a), from 255.97 μm^2^ for control hdFs to 523.18 μm^2^ for sen-hdFs (2.04-fold increase). Nuclear area increased similarly with increasing stiffness for control and sen-hdFs with a 1.26- and 1.25-fold increase from soft (E = 1.7 kPa) to rigid TCP substrates, respectively (Table S4). However, nuclei of sen-hdFs exhibited a noticeably more elongated shape than their non-senescent counterpart on medium stiffness and high stiffness substrates (Fig. 5b). Notably, while control hdFs adopted a more rounded nuclear morphology with increasing stiffness (decreasing aspect ratio), nuclei in sen-hdFs remained elongated (constant aspect ratio).

Given that many of the nuclear structural alterations associated with senescence are driven by changes in the NE [33], we next examined the effects of microenvironmental stiffness on NE integrity (Fig. 5c and d and Fig. S3c and d). To assess NE integrity, we stained for Lamin A (LMNA), a key nuclear lamina (NL) protein that mediates nuclear shape and mechanical stability (Fig. 5c) [51]. Immunostaining of LMNA revealed frequent NE abnormalities in sen-hdFs, including invaginations, protrusions, and wrinkles (Fig. 5c and d). For subsequent quantification, a nucleus was considered defective if it exhibited at least one of these abnormalities. Remarkably, 82.1 % of sen-hdFs nuclei displayed abnormalities on rigid TCP substrates compared to 56.6 % on soft substrates; whereas the percentage in control hdFs was generally low (between 22 and 34 %) and was not significantly affected by substrate stiffness. Previous studies reported on the mechano-responsiveness of LMNA to mechanical stress and cytoskeletal tension [52,53]. Here we, for the first time, observed an increase in LMNA signal with increasing stiffness not only for control hdFs but also for sen-hdFs (Fig. 5c and e and Fig. S3c and e). It was particularly notable that sen-hdFs displayed significantly higher LMNA signals on intermediate-stiff substrates compared to control hdFs, but not on rigid TCP substrates.

The NL senses cytoskeletal forces transmitted through the LINC complex, specifically through SUN-domain proteins [2]. Since SUN2 has been shown to mediate cellular senescence [38,39], we assessed the effects of substrate stiffness on SUN2 levels in the NE of sen-hdFs. Surprisingly, SUN2 was unaffected by changes in stiffness but were consistently reduced in sen-hdFs compared to control hdFs under the same conditions (Fig. 5c and f and Fig. S3c and f). A recent report showed that suppression of SUN2 promotes nuclear decoupling from the cytoskeleton, leading to nuclear softening, delaying the onset of cellular senescence [39]. Thus, the reduction of SUN2 for sen-hdFs found here not only pointed to a weakened nuclear-cytoskeletal coupling but also motivated a closer look at the mechanical properties of their nuclei. Next, we measured nuclear stiffness through nanoindentation using Atomic Force Microscopy (AFM) and found that sen-hdFs grown on rigid plastic (TCP) had significantly softer nuclei (0.71 kPa; Q1: 0.37, Q3: 0.81) compared to that of control hdFs (1.92 kPa; Q1: 1.66; Q3: 1.99), representing a 2.70-fold reduction of the Young's Modulus (Fig. 5g and h ). Taken together, these findings demonstrate an impaired nuclear-cytoskeletal coupling in sen-hdFs that is linked to nuclear softening on rigid TCP substrates. This suggests a surprising mismatch between the adaptation of the cytoskeleton (highly tensioned with large FAs) and the adaptation of the nucleus (reduced stiffness) in response to high-stiffness microenvironments in senescent compared to non-senescent hdFs.

### 2.6 High-stiffness substrates provoke fundamental changes in the nuclear-cytoskeletal mechanical coupling in senescent cells

While we recently observed increased single-cell forces in senescent cells [43], nuclear softening in these cells was unexpected, as it implies reduced mechanical response of the nucleus to increased cytoskeletal forces. To explore this paradox, we examined the geometry of the nucleus from vertical cross-sections of confocal image stacks and assessed nuclear height and volume (Fig. 6a-c). In control hdFs, nuclear height showed a slight, although non-significant, decrease with increasing stiffness from 2.97 μm (Q1: 2.69, Q3: 4.14) on soft substrates to 2.80 μm (Q1: 2.53, Q3: 3.06) on TCP. Sen-hdFs similarly exhibited progressive nuclear flattening with increasing stiffness from soft to intermediate-stiff substrates, with nuclear height decreasing from 2.83 μm (Q1: 2.56, Q3: 3.13) to 2.73 μm (Q1: 2.52, Q3: 2.91), respectively. On high-stiffness TCP, however, this trend was clearly reversed and nuclear height increased markedly to 3.72 μm (Q1: 3.42, Q3: 4.10), suggesting a fundamental change in the mechanical interaction between cytoskeleton and nucleus. To better understand how the geometry of the nucleus changes at this critical point, we calculated the nuclear volume based on measured nuclear height and nuclear area (Fig. 6c and d). Increasing stiffness from soft to TCP substrates led to an increase in nuclear volume from 395.99 μm^3^ (Q1: 305.51, Q3: 455.20) to 579.91 μm^3^ (Q1: 406.62, Q3: 735.89) in control hdFs and from 1347.38 μm^3^ (Q1: 1126.08, Q3: 1682.22) to 2053.05 μm^3^ (Q1: 1723.45, Q3: 2445.40) in sen-hdFs, highlighting a more pronounced increase in nuclear volume in the senescent group. Furthermore, while control hdFs exhibited a consistent positive correlation between nuclear height and nuclear area (R = 0.15) across all substrate conditions, sen-hdFs showed a negative correlation (R = −0.28) on TCP and no correlation on soft and intermediate-stiff substrates (Fig. S4b).

The observed fundamental change in the mechanical interaction between cytoskeleton and nucleus in senescent cells motivated us to investigate the spatial proximity between the actin fibers and the nucleus. Perinuclear actin fiber organization was found to be fundamentally different between hdFs and sen-hdFs with unique stiffness-dependencies (Fig. 6e-g and Fig. S4a). Quantification revealed that the percentage of hdFs exhibiting a perinuclear actin-cap (bundles of aligned actin fibers covering the apical surface of the nucleus), increased from 45.8 % on soft, to 51.1 % on intermediate-stiff substrates, and up to 83.2 % on rigid TCP. Strikingly, sen-hdFs displayed an inverse stiffness-mediated trend, with actin-cap formation decreasing from 77.2 % on soft, to 64.9 % on intermediate-stiff, but down to 37.9 % on rigid TCP substrates. Furthermore, the examination of the arrangement of basal actin fibers in the perinuclear area, revealed the accumulation of short, disorganized actin fibers located at the base of the nuclei in sen-hdFs in response to supra-physiologically rigid substrates.

YAP, and its homolog TAZ, are key mechanotransducers that integrate cytoskeletal tension with nuclear signaling through the LINC complex, and their activity is known to be suppressed in senescence [54,55]. Because YAP/TAZ localization is strongly regulated by actin-generated tension and nuclear compression [20], we next examined whether the alterations in actin-mediated nuclear compression in sen-hdFs affects nuclear YAP localization (Fig. 6h and i and Fig. S5a and b). In control hdFs, nuclear/cytoplasmic (N/C) YAP ratios remained high and largely independent of stiffness. Sen-hdFs, in contrast, exhibited reduced nuclear YAP localization compared to control hdFs on soft substrates and TCP, but showed a clear stiffness-dependent trend: nuclear YAP localization was significantly higher on soft and intermediate-stiff substrates than on TCP, with levels on intermediate stiffness even reaching those of control hdFs.

Collectively, these data show that stiffness modulates actin fiber organization around the nucleus in sen-hdFs, resulting in a perturbed perinuclear actin cap and an accumulation of basal perinuclear actin fibers on high-stiffness substrates. In addition to the reduced proximity between apical actin fibers and the nucleus, the hard-wired mechanical link [56] between the cytoskeleton and the nucleus was weakened in sen-hdFs (Fig. 5f), indicating an impairment in the nucleus’ mechano-regulatory function on high-stiffness substrates. Importantly, the alterations in perinuclear actin organization, and the associated differences in nuclear compression in senescence, may translate into alterations in nuclear mechanotransduction, as indicated by changes in nuclear YAP.

## 3 Discussion

**Fig. 7:**
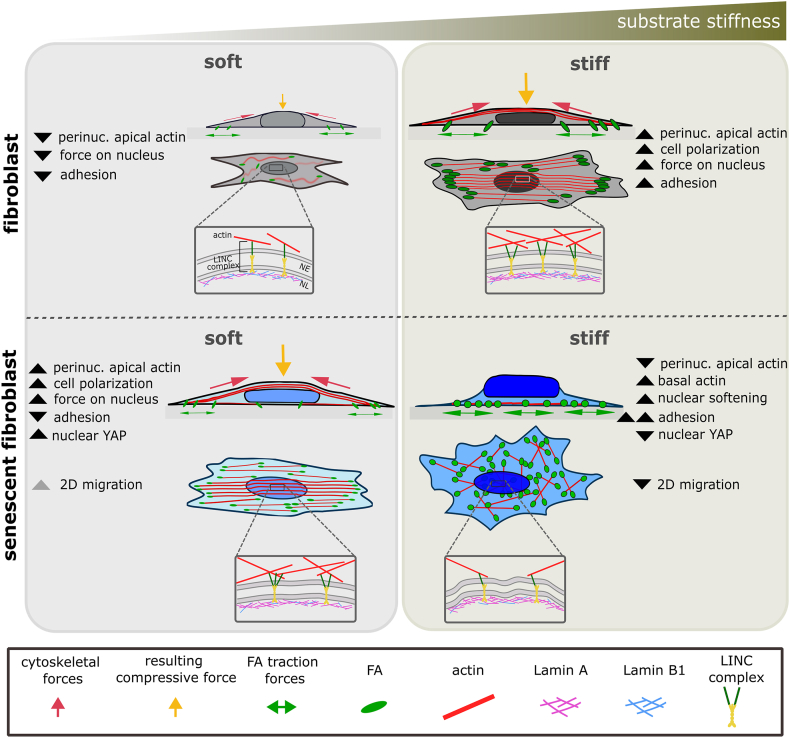
**Proposed model. Substrate stiffness induces actin-mediated nuclear flattening in senescent cells, triggering nuclear-cytoskeletal decoupling once such compression exceeds nuclear tolerance**. Despite pre-existing NE defects, sen-hdFs still present actin caps on soft substrates, adopting a polarized morphology with well-defined stress fibers while the nucleus is being compressed by the perinuclear actin-cap. On soft substrates, the maximum force being applied to the nucleus is inherently limited by the matrix itself. However, as stiffness increases, excessive nuclear compression leads to nuclear decoupling, resulting in nuclear softening and disengagement from the cytoskeletal network. Grey arrows denote proposed mechanisms not yet supported by existing literature.

Using substrate stiffness as a mechano-modulator, we demonstrate that sen-hdFs fail to adequately adapt to mechanical cues. We found that on softer substrates, sen-hdFs are already in a tensional state with a highly compressed nucleus that is confined by perinuclear actin fibers. Increasing stiffness induces pronounced adaptations in cytoskeletal organization and FA assembly, nuclear softening, and leads to a loss of nuclear-cytoskeletal mechanical coupling in sen-hdFs (Fig. 7). Our study reveals that the physiological cellular mechanism of adaptation to substrate stiffness reaches its limit in senescent cells.

Beyond the loss of NE integrity in senescent cells, mainly due to reduced LMNB1, compelling evidence also shows perturbations in the LINC complex [34,37,57]. Our data further suggest that the senescent nucleus lacks sufficient mechanical reinforcement on TCP, as evidenced by nuclear softening. Higher LMNA levels on soft substrates suggest an elevated pre-existing compressive stress applied by the perinuclear actin-cap onto the nucleus in senescent compared to non-senescent cells. At the same time, our observation of reduced SUN2 levels in sen-hdFs indicates an impaired nuclear-cytoskeletal coupling. SUN2 not only regulates SASP factor expression [39] but also protects the nucleus from mechanical stress by cushioning the mechanical link to the cytoskeleton, leading to nuclear softening [18,58]. Here, SUN2 reduction appears independently of substrate stiffness, suggesting a senescence-associated regulatory role rather than a direct mechanical response. Since the presence of an actin-cap strongly relies on the integrity of both the NL and LINC complex [59,60], its disruption in sen-hdFs on TCP may be driven by combined NE impairments, specifically NL defects and SUN2-mediated weakening of nuclear-cytoskeletal coupling. Additionally, LINC complex isoform imbalances, as reported in progeria models [61], are known to affect cell polarity and cytoskeletal organization, and may similarly modulate perinuclear actin dynamics in senescence. However, despite pre-existing NE defects, sen-hdFs still present actin-caps on soft substrates. This suggests that the nuclear decoupling observed on high-stiffness substrates may represent a cellular adaptation to excessive nuclear compression beyond a physiologically tolerated threshold. Soft substrates, in contrast, inherently limit such extensive nuclear compression and allow the mechanical coupling between the nucleus and the cytoskeleton to function physiologically.

As previously reported [44,62], increasing stiffness promotes the formation of well-defined, parallel-aligned actin fibers as well as perinuclear actin bundles in fibroblasts. Our findings show that in sen-hdFs, soft substrates already trigger such parallel actin assembly, leading to cell elongation, actin-cap formation, and nuclear flattening. With increasing stiffness, the coordinated, parallel actin alignment in sen-hdFs is lost and replaced by the formation of individual domains with different fiber orientation, akin to the generally acknowledged senescent pancake-like morphology *in vitro* [28,43,63]. Notably, our study reveals that this specific senescence-associated actin organization is associated with the loss of perinuclear actin bundles and a significant increase in nuclear height, highlighting the role of perinuclear actin in nuclear compression [59,60]. Cell migration represents a key process of maintaining tissue homeostasis and driving wound healing, and it is regulated by perinuclear actin fibers that coordinate the leading-trailing edge dynamics [3,64]. Thus, the loss of the perinuclear actin-cap on TCP may limit cytoskeletal coordination, leading to the observed loss of cell polarization, and potentially explaining the impaired 2D motility of sen-hdFs we recently reported [43]. Such impaired cytoskeletal coordination is expected to have strong implications for the function of senescent cells in tissue healing scenarios. Beyond these functional migratory effects, the loss of the perinuclear actin-cap in sen-hdFs, and the associated loss of nuclear compression, may also influence nuclear mechanotransduction, particularly through changes in YAP localization. In sen-hdFs, YAP regulation appeared stiffness-dependent, with nuclear localization reduced on TCP but increased on soft and intermediate-stiff substrates. This trend is consistent with studies showing that YAP regulation is driven primarily by nuclear compression rather than substrate stiffness alone [20]. By contrast, hdFs did not show stiffness-dependency, in line with their minimal change in nuclear height, suggesting that limited nuclear compression is sufficient to maintain nuclear YAP localization in this context. Importantly, reduced nuclear YAP in sen-hdFs is consistent with previous reports describing suppressed YAP/TAZ activity in senescence [54,55], and our findings suggest that altered nuclear-cytoskeletal coupling and nuclear compression may act as potential modulators of this dysregulation.

Consistent with our previous study showing large, stable FAs in sen-hdFs on rigid TCP [43], we found that FAs in sen-hdFs, in contrast to non-senescent fibroblasts, are less clustered at the cell periphery but more homogenously distributed across the entire cell-substrate contact area. This widespread, hyper-adhesive phenotype could explain the impaired migration of sen-hdFs as a result of reduced FA turnover reported before [65]. The enhanced FA maturation of sen-hdFs on TCP may be influenced by differences in integrin composition and/or mechano-transduction sensitivity [66,67]. Indeed, senescence has been associated with integrin isoform expression differences depending on the model, as we recently reported [43], and with nanoscale rearrangements of core FA components [68], highlighting that FA remodeling in senescence is multifactorial and multifaceted. However, beyond these isoform- and nanoscale-level changes, our data demonstrate that excessive and abnormal FA maturation is restricted to TCP and not observed on softer substrates, where FA size remains comparable to control hdFs. This observation suggests a dysregulated force-feedback loop between adhesion sites and the nucleus under high-stiffness conditions. Given that the senescent nucleus appears disconnected from the cytoskeletal network on TCP, its disengagement from intracellular force-feedback-regulations may contribute to excessive FA maturation. In line with this, LINC protein depletion has been linked to impaired migration, increased nuclear height, and FA accumulation [2,59,69]. Our findings suggest that, beyond a certain stiffness threshold, the loss of nuclear-cytoskeletal coupling occurs naturally within the senescence program. In the absence of the nucleus's key mechano-regulatory function, cytoskeletal organization and tension seem to be more directly controlled by substrate stiffness, leading to pronounced cytoskeletal rearrangements and abnormal adhesion [69].

Our study demonstrates that increasing substrate stiffness leads to progressive nuclear flattening and compression in sen-hdFs, ultimately reaching a mechanical limit in the mechano-adaptation process that provokes nuclear decoupling. We propose that this stiffness-mediated nuclear decoupling in sen-hdFs arises as a form of mechanical relief in response to excessive compressive forces acting on the nucleus, since cells engage in mechanisms to protect nuclear integrity and reduce DNA damage under mechanical stress [18,70]. In line with this, actin-depolymerizing agents have been shown to decrease nuclear stress and rupture by releasing compressive actin forces acting on the nucleus. However, these interventions also disrupt the organization of the perinuclear actin-cap [59,60,71]. An interesting future direction will be to define the stiffness threshold at which decoupling occurs and to test whether a mild pharmacological disruption of the actin cytoskeleton can relieve nuclear compression while preserving sufficient actin cap-mediated coupling.

Unlike in 3D microenvironments where nuclear-cytoskeletal coupling is essential for nuclear positioning and squeezing through the ECM [21,72], nuclear decoupling may be tolerated on flat 2D surfaces, where active repositioning of the nucleus is not required. However, the limited mechanical tolerance of the senescent nucleus to intracellular forces, along with impaired nuclear-cytoskeletal coupling, raises key concerns about their ability to effectively squeeze their nucleus under physiologically relevant 3D constraints [59,[71], [72], [73]], such as tight ECM pores where collagen fibers exert passive compressive forces on the cell. Thus, our study reveals a mechanism that likely extends beyond static 2D culture conditions to more physiological 3D constraints and dynamic environments that challenge nuclear-cytoskeletal integrity. While we do not define a precise stiffness threshold at which nuclear decoupling occurs, our findings establish a broadly applicable principle that lays the groundwork for future studies to both quantify this threshold and determine how and whether this mechanism emerges in more dynamic and physiologically relevant 3D environments.

Across three fibroblast models of senescence, we show that while DNA-damage-induced senescence exhibit the most pronounced changes, the adaptive structural responses remain consistent across all models. This suggests that the observed behaviors may arise from fundamental biophysical alterations intrinsic to the senescent state, rather than from the specific mode of induction. Because such biophysical alterations are consistently reported across diverse senescent models [43,74], and even across cell types [[74], [75], [76], [77], [78]], it is plausible that the altered mechano-adaptation described here is not restricted to dermal fibroblasts but may represent a conserved property of senescent cells more broadly. Additionally, given the broad secretory profile of senescent cells [28,79], which often requires multiple biomarkers for identification [42], biophysical traits may enhance specificity for *in vitro* detection [74]. Although we did not assess phenotypic consequences in this study, recent reports have shown that softer substrates attenuate the senescent phenotype [40,41]. Yet, most of the characterization of senescent cells comes from studies conducted on rigid plastic substrates, conditions that strongly differ from the soft physiological environments where senescent cells can also arise, such as during embryonic development and early tissue repair [[80], [81], [82]]. Such rigid culture conditions may amplify pre-existing defects, exaggerate dysfunction, and therefore, overestimate the *in vivo* cell phenotype occurring in soft environments. However, the here-presented mechanobiological limit associated with sen-hdFs on TCP may not be exclusive to supra-physiological stiff culture settings, as many tissues associated with advanced maturation, such as musculoskeletal tissues, pathological scars, and mineralized matrices [[83], [84], [85]], exhibit stiffness values within the gap between our intermediate-stiff substrate (48.3 kPa) and TCP (GPa-range). Thus, it can be expected that high-stiffness *in vivo* environments like highly fibrotic scar tissue or mineralized tissue impose comparable mechanical challenges on senescent cells as observed here, potentially shaping their behavior and function in pathophysiological tissues.

## 4 Conclusion

In conclusion, our study reveals a limitation in the biophysical adaptation of senescent cells to mechanical cues, bearing consequences for their behavior in high-stiffness microenvironments. Notably, we demonstrate that senescent cells counter excessive nuclear deformations on stiff substrates by disrupting the mechanical link between the actin cytoskeleton and the nucleus resulting in an uncoordinated cytoskeletal organization and suggesting a broader impact on nuclear mechanotransduction. Future studies will be needed to clarify the consequences of this fundamental deviation from physiological mechano-regulation in respect to cell function in 3D environments, particularly nuclear movement and translocation which are key for protrusion dynamics in cell migration [21].

## 5 Materials & methods

### 5.1 Cell isolation & culture

Primary human dermal fibroblasts (hdFs) were isolated from human skin biopsy samples. Cell culture was performed using fibroblast expansion medium consisting of Dulbecco's Modified Eagle Medium (DMEM, 11960-044; Thermo Fischer), fetal bovine serum (FBS 10 % (v/v), S0115; Biochrom AG), Penicillin (100U/ml) and Streptomycin (100 μg/ml) (1 % (v/v) A 2213; Biochrom AG), and nonessential amino acids (NEA, 1 % (v/v), K0293; Biochrom AG). Cells were cultured at 37 °C with 5 % CO2 in a humidified incubator.

### 5.2 Induction of cellular senescence

Generation of tetracycline-inducible constructs (cyclin-dependent kinase inhibitor CDKN2a/p16 over-expression) were previously reported [43] and directly used for this study. Cell cycle arrest was induced in these genetically engineered cells by adding 0.5 μg/ml doxycycline hyclate (D9891; Sigma Aldrich) to the cell culture medium every 2–3 days. Replicative senescence was achieved by long-term serial cell expansion until proliferation was halted (passage number >16). DNA damage was induced to hdFs by stimulation with 1 μg/ml mitomycin C (MMC, M4287; Sigma-Aldrich) for 24h after which, the expansion medium was exchanged and MMC-treated cells were cultured for 14 days prior experiment.

### 5.3 Proliferation analysis

Proliferation rate was quantified using the CyQUANT™ Cell Proliferation Assay (C7026; Thermo Fischer) following the manufacturer's instructions. Cells were seeded at a density of 6500 cells/cm^2^. One day after seeding (day 0), plates were washed once with PBS and frozen at −80 °C. Additional plates were washed and frozen at different time points: 7 and 14 days after seeding. During this period, senescence was induced as described earlier for genetically engineered cells and the cells subjected to DNA-damage. The fluorescence signal was quantified using a plate reader (Infinite PRO; Tecan) with excitation at 480 nm and emission at 520 nm. Cell count was determined based on a standard curve established by a cell pellet with a known number of cells and the raw fluorescence intensities. Population doublings were then calculated by normalizing to the cell count at day 0.

### 5.4 β- Galactosidase activity assay

β-Galactosidase activity was assessed using a commercially available Senescence β-Galactosidase Staining Kit (9860; Cell Signaling Technology) following the manufacturer's instructions. Briefly, cells were plated at of 6500 cells/cm^2^ and kept 14 days in culture. Cells were then fixed with 1X Fixative Solution and stained with β-Galactosidase Staining Solution overnight at 37 °C in a dry incubator. The following day, images were acquired using a standard inverted microscope.

### 5.5 Immunoblotting

Cell lysates were prepared on ice using RIPA buffer (9806; Cell Signaling) supplemented with protease and phosphatase inhibitors (11836153001 and 4906845001; Roche) and 1 % sodium dodecyl sulfate (10 % SDS Solution, 15553027; Thermo Fischer). Prior electrophoresis, 4x Loading Buffer (928–40004; Li-Cor) was added to the lysates and the mixture was denatured at 90 °C for 5 min. Proteins were separated using NuPAGE® 4–12 % gradient Bis-Tris gels (NP0336BOX; Thermo Fischer) in MES SDS Running Buffer (NP0002; Thermo Fisher). The transfer onto nitrocellulose membranes was conducted using XCell II™ Blot Module (Thermo Fischer) for 1h at 30V constant. Membranes were then blocked with TBS Blocking Buffer (927–50000; Li-Cor) and probed overnight with primary antibodies according to the manufacturer's instructions: Phospho-Histone H2A.X (Ser139) (2577; Cell Signaling), GAPDH, (2118; Cell Signaling), p16 INK4A (D7C1M) (80772; Cell Signaling), and P21 Waf1/Cip1 (12D1) (2947; Cell Signaling). The following day, membranes were washed three times with TBS-Tween® 20 (0.1 %, 9127.1; Carl Roth). Secondary antibodies (925–32211 and 925–68070; Li-Cor) were then incubated for 2 h in 3 % BSA/TBS-T. Membranes were then washed three times with TBS-T and scanned using the Odyssey Infrared Imaging System (LiCor). Band signals were quantified by manually contouring of the lanes using the Odyssey Imaging System software. Raw intensity signals were normalized to GAPDH for the respective cell groups.

### 5.6 Polyacrylamide gel substrates preparation

The activation of round coverslips and the preparation of polyacrylamide (PAA) gels of defined stiffness were adapted from previously described methods [86,87]. Briefly, PAA solutions were prepared with 5 % or 12 % acrylamide (161-014; Bio-Rad) and 0.075 % or 0.145 % bis-acrylamide (161-0142; Bio-Rad) respectively, leading to substrate stiffness’ of 1.7 kPa and 48.3 kPa, respectively. The surface of PAA gels was functionalized using UV-activated crosslinker sulfosuccinimidyl 6-(4′-azido-2′- nitrophenylamino)hexanoate (SulfoSANPAH, 22589; Thermo Fischer) at a concentration of 1 mg/ml and irradiation of 2x200s at 10 mW/cm^2^ of 365 nm UV light. PAA gels were then conjugated with monomeric collagen (L7220; Sigma-Aldrich) at a concentration of 38.4 μg/mL in DPBS and incubated overnight at 4 °C. Functionalized collagen-coated PAA and TCP substrates were washed at least 5 times before seeding with cells.

### 5.7 Immunofluorescence staining

Cells on collagen-coated PAA gels were fixed 24 h after seeding with 4 % PFA for 1 h. The fixation was then quenched with 25 mM NH4Cl/PBS for 25 min and samples were rinsed with DPBS. Fluorescent staining was performed for F-actin using phalloidin-Atto 550 (19083; Sigma-Aldrich) or phalloidin 633 (68825; Sigma–Aldrich) to visualize the cytoskeleton of cells and for nuclei (DAPI, D1306; Thermo Fischer). The following primary and secondary antibodies were used: Vinculin (V9131; Sigma-Aldrich), LaminA (ab 8980; abcam), SUN2 (ab 124916; abcam), and YAP (14074; Cell Signaling), Alexa Fluor 488 goat anti-mouse (A32723; Invitrogen), Alexa Fluor 488 donkey anti-rabbit (A32790; Invitrogen), Alexa Fluor 555 goat anti-mouse (A32727; Invitrogen).

### 5.8 Confocal imaging

Confocal microscopy was performed using either a Leica TCS SP5 II confocal laser scanning microscope (Leica Microsystems GmbH), equipped with laser lines and a MaiTai HP multiphoton laser, or a Leica STELLARIS confocal microscope equipped with laser lines. Imaging was conducted with 40X or 63X water immersion objectives at a voxel size resolution of 0.4 μm × 0.4 μm x 1.99 μm or 0.2 μm × 0.2 μm x 0.8 μm (width x length x depth), respectively. Laser power and imaging settings were kept constant across all experimental sessions. Image stacks were recorded by selecting random locations within the gels.

### 5.9 Image analysis

All image analysis was performed in Fiji using custom-built macros.

### 5.10 Actin orientation analysis

The local orientation distribution of the actin fibers was quantified with the maximum intensity projections of the acquired image stacks using the plugin OrientationJ, with a fixed tensor parameter value of 2. The orientation distribution was calculated with respect to the orientation of the cell's major axis (0°). For each individual analyzed cell, circular variance was computed using a custom-built MATLAB script. This script extracted the OrientationJ-derived angular distributions of actin fiber alignment within each cell (orientation angles and corresponding pixel frequencies) and calculated its circular variance. A value of 0 indicates perfect alignment, while a value of 1 reflects random or isotropic of actin fibers.

### 5.11 Focal adhesion analysis

A maximum intensity projection of the planes containing the focal adhesion was selected from each stack of the acquired images. Background subtraction and contrast enhancement was applied to the image. Focal adhesions were then binarized. The Analyze Particle function was used to extract the shape parameters and number of focal adhesions using a size exclusion criteria of >0.073 μm^2^. The number of focal adhesions located at the periphery of cells was determined by shrinking the contour of the cell by 35 % and counting the amount of focal adhesions in the region between the original cell contour and the shrank one.

### 5.12 LaminA and Sun2 analysis

For each analyzed cell, a nuclear mask was created based on the DAPI signal using a constant threshold. The mask for the nuclear envelope was then generated by shrinking the contour of the DAPI ROI by one pixel. The middle plane of the nuclear envelope was manually selected from each stack of the acquired images. The integrated density of the protein of interest (LMNA or SUN2) within the ROI of the nuclear envelope was then measured. To account for nuclear size differences, this intensity was normalized to the perimeter of the nuclear envelope of each analyzed cell.

### 5.13 Nuclear height and approximate volume

Nuclear height was quantified from confocal z-stack images of LMNA-stained cells. For each nucleus, the nuclear area was extracted from maximum projection and the vertical distance between the upper and lower boundaries of the LMNA was measured in both the XZ and YZ orthogonal views. The average of these two measurements was taken as the nuclear height. Nuclear volume was approximated by modeling the nucleus as an ellipsoid using the measured area and height. Nuclear volume reconstruction and rendering was performed using Imaris v9.9.1.

### 5.14 Nuclear to cytoplasmic (N/C) YAP ratio

Nuclei were segmented from the DAPI signal, and cytosolic regions were defined from the actin channel based on the identified nuclei. YAP mean intensity was quantified in nuclear and predefined cytosolic ROIs from maximum projections and a nuclear-to-cytoplasmic ratio was calculated per cell.

## 6 Atomic force microscopy

Atomic force microscopy was performed on cells using a CellHesion®200 AFM system (JPK/Bruker, Berlin, Germany) equipped with an inverted Zeiss Axio Observer 3 microscope. Live-cell images were captured using a 25X objective. Cells were cultured for 24 h before AFM-imaging and rinsed with pre-warmed DPBS to avoid laser interference or tip contamination. Before probing a cell, a scan grid was created that included part of the (1) nucleus, (2) cytoskeleton, and (3) plastic surface to use as a reference when calculating relative heights. Cells were probed with a 1 μm spherical tip cantilever using a standard approach with 1 nN maximum indentation force. Data processing was performed with the JPK Data Processing software. Force-distance curves for each individual indentation point within the scanned grid were generated using the Hertz/Sneddon model. By using the overlay between the image of the cell and the scanned grid, the individual pixels within the nucleus were selected and an average nucleus's Young's Modulus was calculated for each probed cell.

## 7 Statistics

All plots were generated using the OriginPro 2020 (OriginLab Corporation) software. Data is presented as mean values and corresponding standard deviation. Box plots are shown as boxes with 25 % lower and 75 % upper limit. Individual data points are overlaid with the bar chart or box plot as black dots. Statistical significance was assessed using Mann-Whitney-U-tests. Multiple comparisons were adjusted using Bonferroni correction. Significance levels are indicated as: ∗p < 0.05, ∗∗p < 0.01, and ∗∗∗p < 0.001. For comparisons within same cell group, significance is indicated using asterisks accompanied by connecting lines. For comparisons between control cells (hdFs) and senescent hdFs (p16, RS, or MMC) under the same substrate condition, significance is indicated by asterisks placed directly above the relevant bar or box.

## CRediT authorship contribution statement

**Mina Sohrabi Molina:** Writing – review & editing, Writing – original draft, Visualization, Methodology, Investigation, Funding acquisition, Formal analysis, Data curation, Conceptualization. **Erik Brauer:** Writing – review & editing, Validation, Supervision, Resources, Project administration, Methodology, Funding acquisition, Conceptualization. **Rebecca Günther:** Investigation, Methodology. **Stephanie Diederich:** Investigation. **Ansgar Petersen:** Writing – review & editing, Validation, Supervision, Resources, Project administration, Funding acquisition, Conceptualization.

## Declaration of competing interest

The authors declare that they have no known competing financial interests or personal relationships that could have appeared to influence the work reported in this paper.

## Data Availability

Data will be made available on request.
