# Supplementary material for: Increased substrate stiffness disrupts nuclear-cytoskeletal mechanical coupling in senescent cells
Source: Mater Today Bio. 2025 Oct 27;35:102472. doi: 10.1016/j.mtbio.2025.102472 (PMC12621461; doi:10.1016/j.mtbio.2025.102472)
Supplement: Multimedia component 1 [file mmc1.pdf]

Supplementary Figures

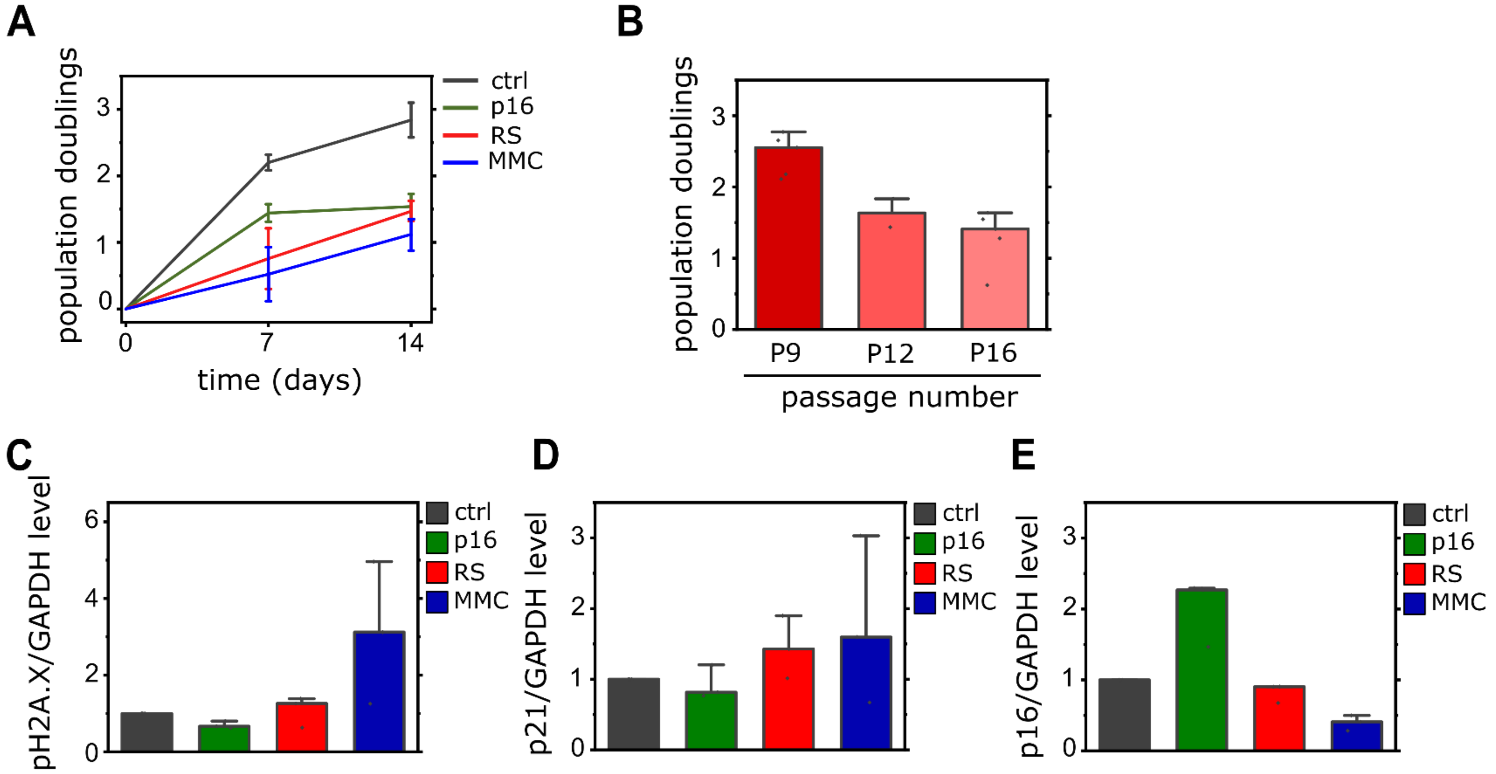

Figure S1: (A) Population doublings of sen-hdFs (p16, RS, and MMC) over 14 days in culture (N=4-5). **(B)** Population doublings of hdFs by passage number (N=2-5). Quantification of WB band intensities of sen-hdFs normalized to loading control GAPDH of **(C)** DNA damage marker phospho-H2A.X, **(D)** cell cycle inhibitors, p21Cip and **(E)** p16Ink4a (N=2-3).

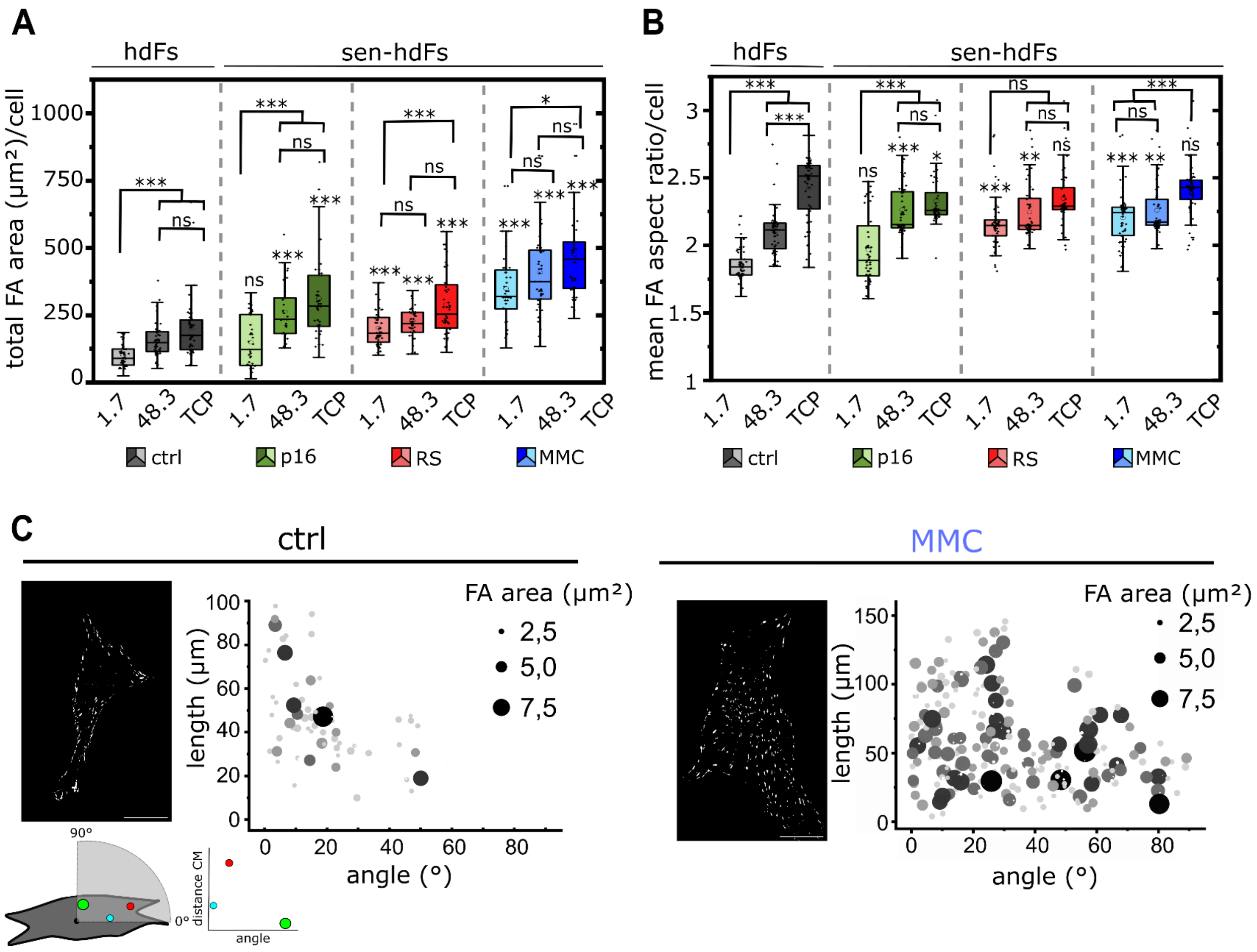

Figure S2: (A) Quantification of total FA area per cell and **(B)** mean FA aspect ratio per cell of hdFs (ctrl) and sen-hdFs (p16, RS and MMC) cultured for one day on collagen-coated stiffness-varying substrates: soft (E = 1.7 kPa), intermediate (E = 48.3 kPa), and rigid TCP (E ~ 1 GPa) (N≥50). **(C)** Bubble plots showing FA distribution in a representative control cell (hdF) and MMC-treated cell as a function of FA position relative to the cell center. Each bubble represents an individual FA, with its angular position (°) and distance from the cell center (length). Bubble size indicates the area of the FA (µm²). Scale bar 50 µm.

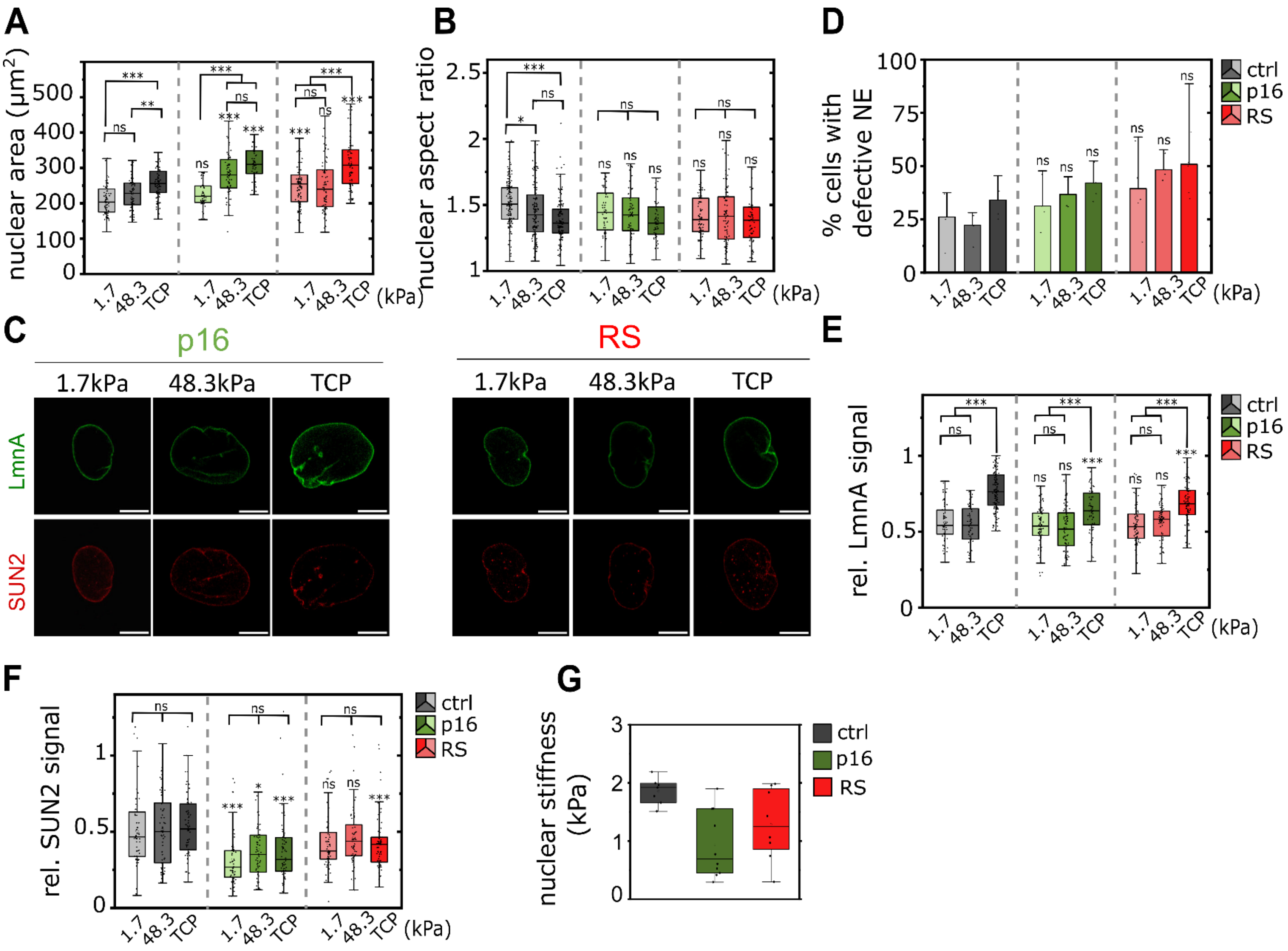

Figure S3. (A) Quantification of changes in nuclear area and **(B)** nuclear aspect ratio of hdFs (ctrl) and senescent hdFs (p16 and RS) cultured for one day on collagen-coated stiffness-varying substrates: soft (E = 1.7 kPa), intermediate (E = 48.3 kPa), and rigid TCP (E ~ 1 GPa). **(C)** Representative immunofluorescence staining of LMNA (green) and SUN2 (red) in the nuclear envelope of hdFs (ctrl) and senescent hdFs (p16 and RS). **(D)** Quantification of percentage of cells with abnormal nuclear envelope in hdFs and senescent hdFs (p16 and RS) cultured on stiffness-varying substrates. Each data point represents an individual experiment (N=4-6) in which at least 30 nuclei where analyzed per condition. **(E)** Quantification of relative nuclear envelope LMNA signal and **(F)** SUN2 signal in response to stiffness-varying substrates (N≥60). **(G)** Quantification of Young's Modulus of the nucleus of hdFs (ctrl) and senescent hdFs (p16 and RS) cultured for one day on collagen-coated TCP. Each data point represents an individual cell (N≥7) cells from 2 independent AFM experiments. Scale bar 10 µm.

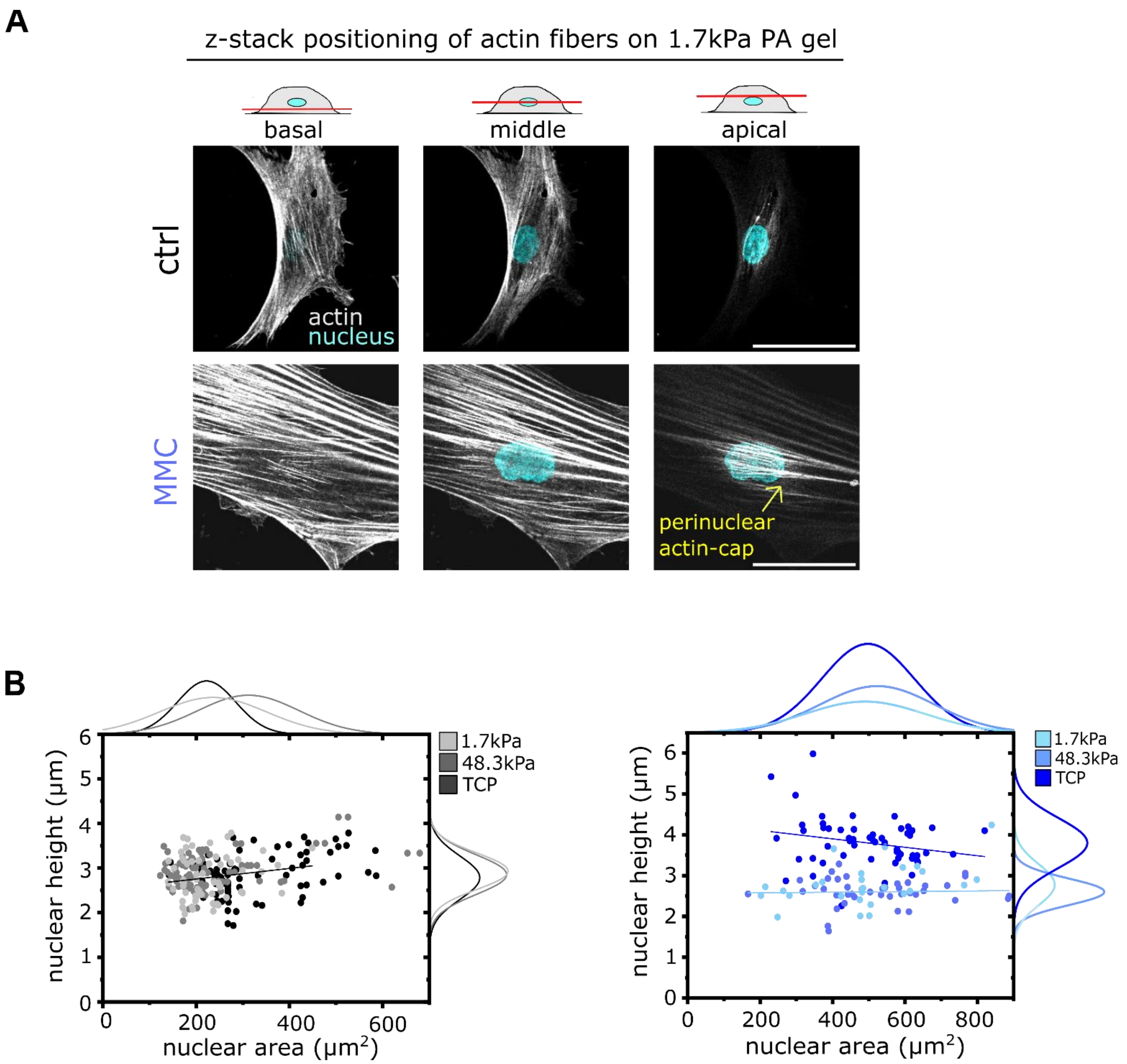

Figure S4. (A) Representative immunostained images of hdFs (ctrl) and senescent hdFs (MMC) cultured on collagen-coated soft gel (E=1.7kPa) showing confocal sections of the actin fiber network around the perinuclear area at the basal (left), mid-height (middle), and apical surface (right). Cells were stained for actin (gray) and LMNA (cyan). Apical surface (right) displays lack of actin cap formation in control hdFs and prominent organization of perinuclear actin fibers in sen-hdFs. **(B)** Correlation between nuclear height and nuclear area of hdFs (ctrl) and senescent hdFs (MMC) cultured for one day on collagen-coated stiffness-varying substrates: soft (E = 1.7 kPa), intermediate (E = 48.3 kPa), and rigid TCP (E ~ 1 GPa). Scale bar 50 μm.

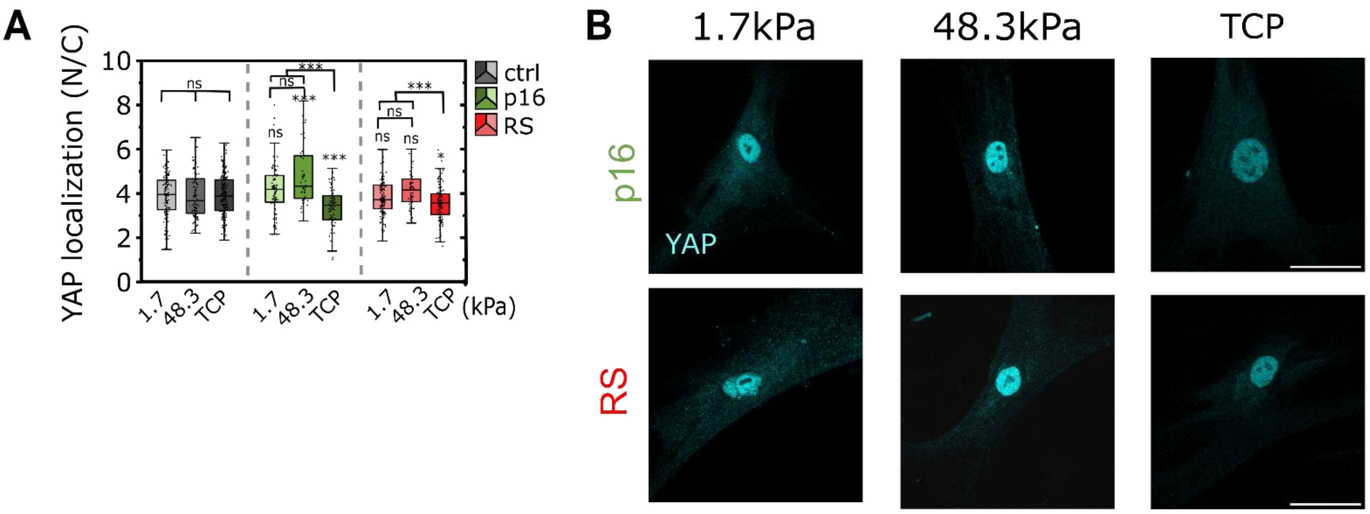

Figure S5. **(A**) Quantification of Nuclear to Cytoplasmic (N/C) YAP ratio of hdFs (ctrl) and senescent hdFs (p16 and RS) cultured for one day on collagen-coated stiffness-varying substrates: soft (E = 1.7 kPa), intermediate (E = 48.3 kPa), and rigid TCP (E ~ 1 GPa). **(B)** Representative immunostained images of senescent hdFs (p16 and RS) cultured on the stiffness-varying substrates. Cells were stained for YAP (cyan). Each data point represents an individual cell (N≥60 cells) from 3 independent experiments Significance levels indicate: * $p < 0.05$, ** $p < 0.01$, and *** $p < 0.001$. For comparisons within same cell group, significance is indicated using asterisks accompanied by connecting lines. For comparisons between control cells (hdFs) and senescent hdFs (p16 and RS) under the same substrate condition, significance is indicated by asterisks placed directly above the box. Scale bar 50 µm.

Table S1. Cell area on soft and TCP and corresponding fold change

Cell Group	Median cell area on soft substrate ($\times 10^4$ μm^2)	Median cell area on TCP ($\times 10^4$ μm^2)	Fold Change
**ctrl**	0.57	0.69	1.21
**p16**	0.66	1.34	2.03
**RS**	0.72	1.22	1.69
**MMC**	1.47	2.05	1.39

Table S2. Cell aspect ratio on soft and TCP and corresponding fold change

Cell Group	Median cell aspect ratio on soft substrate	Median cell aspect ratio on TCP	Fold Change
**ctrl**	2.64	2.26	1.17
**p16**	2.58	1.93	1.34
**RS**	2.63	2.08	1.26
**MMC**	2.58	1.96	1.32

Table S3. Mean FA size (per cell) on soft and TCP and corresponding fold change

Cell Group	Median FA size on soft substrate (μm^2)	Median FA size on TCP (μm^2)	Fold Change
**ctrl**	0.98	1.45	1.48
**p16**	0.95	1.80	1.90
**RS**	1.00	1.99	1.99
**MMC**	0.99	2.07	2.09

Table S4. Nuclear area on soft and TCP and corresponding fold change

Cell Group	Median nuclear area on soft substrate (μm^2)	Median nuclear area on TCP (μm^2)	Fold Change
**ctrl**	203.49	255.97	1.26
**p16**	222.25	308.48	1.39
**RS**	254.61	308.02	1.21
**MMC**	417.82	523.18	1.25
